# No evidence for increased extinction proneness with decreasing effective population size in a parasitoid with complementary sex determination and fertile diploid males

**DOI:** 10.1186/1471-2148-10-366

**Published:** 2010-11-26

**Authors:** Jan Elias, Silvia Dorn, Dominique Mazzi

**Affiliations:** 1 ETH Zurich, Institute of Plant, Animal and Agroecosystem Sciences/Applied Entomology, Schmelzbergstrasse 9, CH-8092 Zurich, Switzerland

## Abstract

**Background:**

In species with single locus complementary sex determination (sl-CSD), the sex of individuals depends on their genotype at one single locus with multiple alleles. Haploid individuals are always males. Diploid individuals are females when heterozygous, but males when homozygous at the sex-determining locus. Diploid males are typically unviable or effectively sterile, hence imposing a genetic load on populations. Diploid males are produced from matings of partners that share an allele at the sex-determining locus. The lower the allelic diversity at the sex-determining locus, the more diploid males are produced, ultimately impairing the growth of populations and jeopardizing their persistence. The gregarious endoparasitoid wasp *Cotesia glomerata *is one of only two known species with sl-CSD and fertile diploid males.

**Results:**

By manipulating the relatedness of the founders, we established replicated experimental populations of the parasitoid *C. glomerata *differing in their genetic effective size, and thus in allelic richness at the sex-determining locus and in the expected magnitude of diploid male production. Our long-term survey of population welfare and persistence did not provide evidence for increased proneness to population extinction with decreasing initial genetic effective population size. Most recorded surrogates of fitness nevertheless decayed over time and most experimental populations eventually went extinct, suggesting that the negative effects of inbreeding outweighed any premium from the fertility of diploid males.

**Conclusions:**

The fertility of diploid males may have evolved as an adaptation prompted by the risk of extinction looming over small isolated populations of species with sl-CSD. However, fertility of diploid males does not negate the costs imposed by their production, and although it may temporarily stave off extinction, it is not sufficient to eradicate the negative effects of inbreeding.

## Background

Humanity derives various benefits from services provided by a large number of populations of species [1], such as the sustainable development of agricultural crops [2]. However, anthropogenic activities have accelerated the global decline of biodiversity [3], leading to detrimental consequences on human welfare [4]. Many important ecosystem providers are endangered by habitat destruction, invasive species, overexploitation, or climate change [5] and have small or declining populations [6] with genetic effective population sizes orders of magnitude smaller than the actual demographic population size [7].

In small populations, the loss of heterozygosity necessarily occurs as a consequence of inbreeding and of the random loss of alleles through genetic drift [8]. Following genetic drift, some deleterious alleles will be lost, while others will become fixed, ultimately leading to a genetic load [9]. Under inbreeding, i.e. when relatives mate more frequently than expected by chance, allele frequencies remain unchanged, but the proportion of homozygous genotypes is increased. Inbreeding depression [10,11] is amply documented to lower the fitness of offspring of closely related individuals in numerous animal and plant species [11-16], and may ultimately enhance the risk of population extinction [17]. In haplodiploid insect species, in which fertilized eggs develop into females while unfertilized eggs develop into males, inbreeding depression is often assumed to be less severe than in diploid species, because recessive deleterious and lethal mutations are purged in haploid males [18-20]. However, haplodiploids may also suffer from inbreeding depression, especially in female-limited traits such as fecundity and sex ratio [10,19,21].

In haplodiploid Hymenoptera, inbreeding depression may further be exacerbated in species with single locus complementary sex determination (sl-CSD). Under sl-CSD, which has been demonstrated in more than 60 species of bees, ants and wasps and is presumed to be ancestral in the Hymenoptera [22-24], sex is determined by one single locus with multiple alleles [25-27]. While haploid (hemizygous) individuals are always male, diploid individuals are female when heterozygous and male when homozygous at the sex-determining locus. Diploid males predominantly occur under inbreeding and are typically regarded as a genetic load for populations due to their low viability [27-29], sterility [30], or inability to mate [31]. Diploid males may also mate properly but produce triploid, sterile offspring [32-34]. Furthermore, the control of females over progeny sex ratio through the fertilization process of their eggs is compromised when diploid males are produced at the expense of females [35].

In species with sl-CSD, the production of unviable or effectively sterile diploid males is theoretically predicted to make small, isolated populations particularly prone to extinction [36]. Thus, species with sl-CSD are expected to have evolved mechanisms to counteract the genetic load from diploid males, a particular form of inbreeding depression [25]. Inbreeding is generally regarded to fuel the evolution of inbreeding avoidance mechanisms [37], as documented in behavioural studies of several hymenopteran species [38-41]. Within the braconid genus *Cotesia*, a variety of peculiar mechanisms alleviating the diploid male load are known. *Cotesia flavipes *and *C. sesamiae *circumvent the diploid male load altogether by not exhibiting CSD [42]; *C. vestalis *evolved multiple locus complementary sex determination (ml-CSD), significantly lowering the production of diploid males [32,43], and *C. glomerata *evolved diploid male fertility, enabling diploid males to sire reproductive diploid daughters [44]. So far, reproductive diploid males are known from only one other species with sl-CSD [45].

An earlier experimental manipulation of the genetic effective size of populations via the relatedness of founders, and thus of the pool of alleles passed to the next generation irrespective of census size, revealed the expected effect on the extinction proneness of populations of the annual evening primrose *Clarkia pulchella *[46]. Here, we have monitored replicated experimental populations of the gregarious endoparasitoid wasp *C. glomerata *established with small, medium, or large genetic effective population sizes. Small isolated haplodiploid populations with sl-CSD and sterile diploid males have theoretically been shown to be more prone to extinction than diploid populations and haplodiploid populations without sl-CSD [36], but the extent to which reproductive diploid males affect the welfare and persistence prospects of populations of species with sl-CSD remains unknown. More diploid males are produced in populations with smaller genetic effective sizes and thus poor allelic diversity and a high likelihood of matings between partners sharing a common allele at the sex-determining locus [47]. Therefore, differences in fitness and survival are expected among populations initiated with different genetic effective sizes, unless the fertility of diploid males offsets the genetic load of their production. The production of diploid males leads to a distortion of the population sex ratio, and thus to a surplus of males [18], and females inseminated by diploid males produce fewer daughters than females inseminated by haploid males [44]. Hence, despite the fertility of diploid males, a genetic load is nevertheless expected. Accordingly, we predicted experimental populations initiated with the largest genetic effective size to perform best and persist the longest and experimental populations initiated with the smallest genetic effective size to perform worst and go extinct the soonest. Experimental populations initiated with intermediate genetic effective population size were predicted to rank in between. To our knowledge, this is the first work on an animal species to experimentally manipulate the genetic effective population size independently of the census size of the founder population, and to assess the long-term effects of the manipulation on extinction proneness. An improved understanding of the population dynamics of haplodiploids with sl-CSD is imperative for improved conservation measures to safeguard the abundance and diversity of providers of keystone services in natural and agricultural systems.

## Methods

### Field sampling and laboratory rearing

*Cotesia glomerata *parasitoids were sampled four times each in the summers of 2006, 2007 and 2008 in a large cabbage-growing area near Unter-Stammheim, Zurich, Switzerland (47°38' N, 8°46' E, 420 m AMSL). For each collection, at least 35 potted Brussels sprout plants (*Brassica oleracea *var. *gemmifera*) infested with 20-40 second instar caterpillars of the host, *Pieris brassicae*, were exposed to parasitism in the field for two to three days. The *P. brassicae *caterpillars parasitized in the field were returned to an insectary and fed on Brussels sprout plants at a temperature of 21 ± 1°C, 60% relative humidity (r.h.) under a light dark regime of 16 h:8 h (16L:8D). After parasitoid larvae egress from the host, they spin cocoons for pupation. Cocoon clusters were kept at 15°C, 70% r.h., 16L:8D. Upon emergence, adult parasitoid wasps were reared in insect cages (30 × 30 × 30 cm) and kept at 15°C, 70% r.h., 16L:8D. To maintain the colony, second instar *P. brassicae *caterpillars were placed into the wasp rearing cages to be parasitized, and the cycle started anew. The above rearing conditions were maintained throughout the experiment.

### Experimental populations with different initial genetic effective population sizes

To obtain full siblings of *C. glomerata *for the initiation of experimental populations, two controlled preliminary generations were bred. In the first preliminary generation, parasitoid cocoons were individually isolated in order to obtain virgin females and males, thus excluding multiply inseminated females and sperm-depleted males. In the second preliminary generation, pairs of virgin females and males from different field collections were mated, thus promoting outbreeding. Females were individually given hosts for oviposition, excluding non-self superparasitism [48]. For three consecutive years, every year we set up four experimental populations for each of three different treatments with either small, medium, or large initial genetic effective population size. Thus, overall, 36 experimental populations were established (twelve replicates for each of the three treatments). Each experimental population was founded with twelve randomly allocated wasps (six males and six females). Individuals derived from the same cocoon cluster were only used for a maximum of two experimental populations in different treatments. Experimental populations with small initial genetic effective population size were established with twelve siblings (six males and six females). Groups of siblings bear either two or three sex alleles, depending on whether their parents were matched or unmatched at the sex-determining locus. Earlier simulations showed that even in the extreme case of inbred lines, each harbouring as few as two alleles, a small number of lines can maintain a surprisingly high allelic richness, namely at least as many unique alleles as there are lines [49]. The founders of our populations were, however, drawn from a large genetic pool, hence we estimate a likely minimum number of two unique alleles in the progeny of each female. Experimental populations with medium initial genetic effective population size were established with three sets of four siblings (three times two brothers and two sisters). Thus, up to nine sex alleles were present in experimental populations with medium initial genetic effective size. Experimental populations with large initial genetic effective population size were established with six brother-sister pairs (six times two opposite-sex siblings). Accordingly, up to eighteen sex alleles were present in experimental populations with large initial genetic effective size. Typical estimates of the diversity of sex alleles in natural populations range between nine and twenty [25], matching the range encompassed by our treatments. The magnitude of inbreeding in the natural source population of the used wasp is not expected to affect the set up of our experimental populations, as inbreeding does not alter allele frequencies, but rather rearranges alleles into disproportionately frequent homozygous combinations [50]. The presence of diploid males amongst the founders does not affect allelic diversity at the sex-determining locus, as diploid males harbour two copies of the same allele. Any systematic bias in the representation of diploid males was avoided by randomly assigning founders to treatments.

### Maintenance of experimental populations

To establish the experimental populations, wasps of the founder generation were given 60 hosts to parasitize during 24 hours. Thereafter, each experimental population was presented with 60 hosts for 10-15 minutes in every generation three to five days after emergence of the first adult wasp. For each experimental population and in each generation, ten cocoon clusters were used to maintain individual experimental populations, thus keeping the census size of experimental populations well above a hundred throughout, while five more random clusters were used for measurement of fitness surrogates. Superfluous cocoon clusters were discarded. Experimental populations were surveyed for a maximum of seven generations (i.e. ca seven months) or until extinction. Experimental populations went extinct whenever they fell below an arbitrary threshold of five cocoon clusters (as required for fitness measurements) egressing out of the 60 hosts.

### Surrogate measures of fitness

The brood size (number of cocoons in each cluster), number of emerged wasps and hatching success (proportion of cocoons out of which adults emerged) were determined. Furthermore, we recorded the time in days from oviposition until egression of larvae from their host and from oviposition to the emergence of the first and of the last adult wasp. To obtain the sex ratio (proportion of males), emerged adults were sexed and counted. The right hind tibia length of three randomly chosen males and females was measured at 50× magnification under a Wild M5A stereomicroscope (former Wild Heerbrugg AG, Switzerland) equipped with an ocular micrometer (scale = 0.016 mm). The length of the right hind tibia is a significant correlate of body mass (own unpublished data).

### Statistical analysis

All data were analyzed using R software [51]. To determine whether the initial genetic effective population size had an effect on the extinction probability of experimental populations, a Kaplan Meier survival analysis with a non-constant hazard based on the Weibull distribution was conducted using the survival package in R [52]. Fitness data were analyzed using linear mixed models with random slopes. Mixed model analyses were performed using the R packages nlme [53] and lme4 [54]. The experiment ran for three years, thus year was entered as a blocking factor. Treatment (initial genetic effective population size) was modelled as a fixed factor and generation as a covariate.

## Results

**Figure 1:**
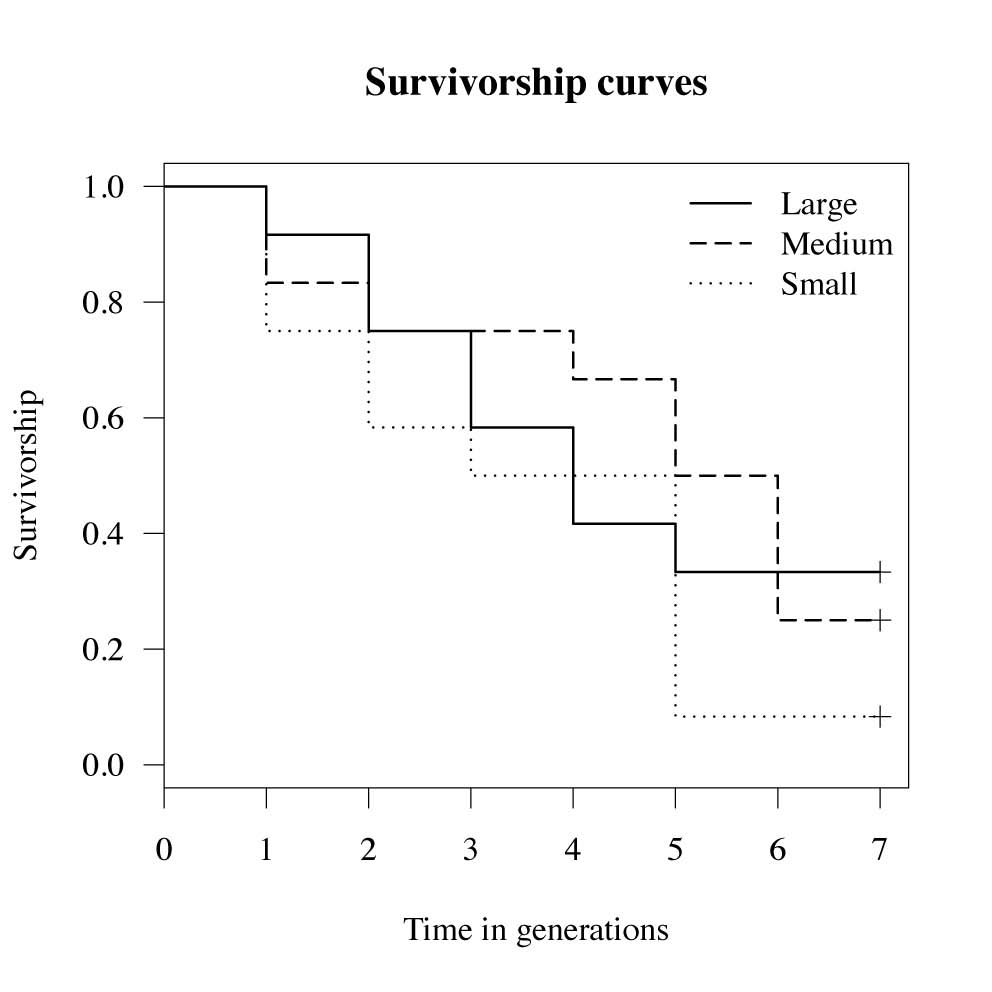
**Population survival**. Survivorship curves of the twelve experimental *C. glomerata *populations for each of three different initial genetic effective population sizes

Out of the twelve experimental *C. glomerata *populations established for each of the three initial genetic effective population size treatments, eight with large, nine with medium and eleven with small initial genetic effective population size went extinct before the seventh generation. Despite raw figures in agreement with the direction of our prediction, the survival analysis did not reveal any significant differences between populations initiated with different genetic effective sizes. As illustrated in Figure 1, the survivorship curves cross and swap ranks repeatedly. Half of the experimental populations with small initial genetic effective population size went extinct after three generations and the survival analysis predicted extinction for such populations after four generations. Experimental populations with medium and large initial genetic effective population size were predicted to go extinct after six generations.

Surrogate measures of fitness indicated little differences between populations with different initial genetic effective sizes (Additional file 1). A significant treatment by generation interaction on the time to the emergence of the first and of the last adult wasp significantly distinguished populations with large initial genetic effective population size from the ones with medium initial genetic effective population size (P < 0.05), but not from the ones with small initial genetic effective population size.

Generation had significant negative effects on brood size (decreasing over time; P < 0.01), emerged female and male adults (decreasing over time; P < 0.01), sex ratio (i.e. proportion of males, increasing over time; P < 0.05) and time to egression of parasitoid larvae from hosts (increasing over time; P < 0.01), indicating deleterious effects of inbreeding, regardless of treatment. Hatching success and tibia length of males and females remained unaffected by either treatment or generation effects.

## Discussion

Our long-term survey of replicated experimental populations initiated with different genetic effective population sizes aimed to investigate fitness and persistence of isolated populations of a gregarious parasitoid with sl-CSD and fertile diploid males. A small genetic effective population size is tantamount to low variability at the sex-determining locus, leading to high occurrence of matings between partners sharing an allele at the sex-determining locus, and thus frequent production of diploid males [18,25]. Diploid male production in species with sl-CSD is theoretically predicted to impair population welfare and to promote population extinction [36]. We found no evidence that the extinction proneness of experimental populations was affected by their initial genetic effective size and, by proxy, the magnitude of diploid male production [47]. Thus, in isolated populations of *C. glomerata*, the risk of extinction was largely independent of the initial genetic effective population size. Furthermore, we found no evidence for an effect of the initial genetic effective population size on surrogate measures of fitness, with the exception of developmental time to adult emergence. Individual wasps emerged earlier in experimental populations with large initial genetic effective size than in experimental populations with medium initial genetic effective size. However, experimental populations with small initial genetic effective size did not significantly differ from the other treatments, undermining the biological significance of the effect. The documented increase in the proportion of males over time is likely indicative of increased diploid male production and hence of inbreeding [18]. The current finding that the risk of extinction is not magnified in populations with small initial genetic effective population size intriguingly suggests that the reproductive fate of diploid males crucially affects the speed with which the postulated vortex [36] pushes population of species with sl-CSD towards extinction.

When populations face the threat of extinction, adaptations may counter the rate of decline and rescue populations, as was recently shown in yeast populations exposed to lethal concentrations of salt [55]. A prerequisite for this rapid evolution is that populations harbour sufficient genetic variability. Favourable genotypes, on which selection can act quickly, may occur at very low frequency [56]. When small, isolated populations of hymenopterans with sl-CSD face an elevated threat of extinction due to a diploid male load [36], selection will act on traits or behaviours that counteract the diploid male load. Species with sl-CSD may have a very low frequency of reproductive diploid males [30]. When the occurrence of typically unviable or sterile diploid males is increased under systematic inbreeding [22,25], selection may act on the otherwise rare fertile diploid males. Thus, commonly occurring fertile diploid males in *C. glomerata *[44] and *Euodynerus foraminatus *[45] may be an adaptation prompted by the pressure of the extinction vortex. Nevertheless, extinction events occurred recurrently in the course of our experiment (28 out of 36 experimental populations fell below our set extinction threshold) and eight out of eleven surrogate measures of fitness decayed over time. We conclude that in isolated populations, the detrimental effects of inbreeding eventually overshadow any beneficial effect of diploid male fertility, irrespective of the initial genetic effective population size and despite optimized rearing conditions.

To understand the deleterious consequences of inbreeding is of applied significance as well. Biological control always relies on mass-reared control agents for release in areas of pest infestation. Many of the taxa used as biological control agents have sl-CSD [24]. The relatively high rate of biological control failures was associated with the rate of sterile diploid males produced under inbreeding [57]. Species with fertile diploid males [44,45] should, therefore, have better prospects of success as biological control agents reproducing and establishing populations in the field. However, even in such species, mass-reared isolated populations maintained in the laboratory may be at risk of extinction. Any gene flow could mitigate this risk, as even low dispersal rates decisively enhance population survival [58]. In mass rearing of biocontrol agents, regular introduction of field-collected specimens could simulate such gene flow and boost the success of biological control programs. We speculate that the lack of dispersal opportunities and thus of genetic exchange between populations was one important reason for the extinction of many of our experimental populations.

## Conclusions

Fertility of diploid males in species with sl-CSD [44,45] may be a rare example of an evolutionary adaptation alleviating the extinction threat posed by a peculiar mode of sex determination in natural populations. Experimental populations initiated with different genetic effective size were similarly prone to extinction, regardless of the extent to which they were burdened by the production of diploid males. However, frequent extinction events and obvious signs of inbreeding depression highlight the vulnerability of small isolated populations. Anthropogenic changes of climate and landscape may eventually drive even apparently well adapted taxa, such as valued parasitoids of common farm pests, to extinction. Thus, conservation measures for species facing an immediate risk of extinction and close monitoring of species that occupy vulnerable habitats must remain an essential part of our effort to safeguard what remains of biodiversity.

## Authors' contributions

DM conceived of the research question; JE, SD and DM designed and coordinated the experiment, discussed the interpretation and wrote the manuscript; JE performed the collection of the data and conducted the statistical analyses. All authors read and approved the final manuscript.

## Supplementary Material

Additional file 1 **Measures of fitness**. Mean ± standard deviation for all surrogate measures of fitness given for experimental populations with different initial genetic effective population sizes (small, medium and large). Click here for file
