# Supplementary material for: No evidence for increased extinction proneness with decreasing effective population size in a parasitoid with complementary sex determination and fertile diploid males
Source: BMC Evol Biol. 2010 Nov 26;10:366. doi: 10.1186/1471-2148-10-366 (PMC3004921; doi:10.1186/1471-2148-10-366)
Supplement: Additional file 1 — Measures of fitness. Mean ± standard deviation for all surrogate measures of fitness given for experimental populations with different initial genetic effective population sizes (small, medium and large). [file 1471-2148-10-366-S1.DOC]

**Table 1**

| Fitness measures | Generation 1 | Generation 2 | Generation 3 | Generation 4 | Generation 5 | Generation 6 | Generation 7 |
| --- | --- | --- | --- | --- | --- | --- | --- |
| Brood size |  |  |  |  |  |  |  |
| Small | 39±16 | 42±32 | 31±37 | 23±27 | 14±16 | 4±13 | 5±19 |
| Medium | 42±16 | 37±23 | 38±29 | 31±25 | 26±29 | 22±25 | 15±29 |
| Large | 45±19 | 46±23 | 40±29 | 35±35 | 21±30 | 15±23 | 14±23 |
| Emerged wasps |  |  |  |  |  |  |  |
| Small | 26±15 | 21±21 | 21±24 | 14±20 | 10±12 | 3±9 | 4±15 |
| Medium | 26±13 | 17±16 | 18±19 | 15±14 | 12±18 | 12±14 | 6±12 |
| Large | 31±18 | 22±16 | 23±21 | 21±23 | 10±15 | 7±13 | 8±13 |
| Emerged males |  |  |  |  |  |  |  |
| Small | 10±10 | 12±14 | 11±15 | 8±12 | 6±9 | 2±7 | 2±8 |
| Medium | 12±12 | 9±10 | 10±11 | 8±9 | 6±11 | 6±8 | 4±10 |
| Large | 11±7 | 12±10 | 12±15 | 12±15 | 5±9 | 4±8 | 5±10 |
| Emerged females |  |  |  |  |  |  |  |
| Small | 15±12 | 8±11 | 10±12 | 6±9 | 4±7 | 1±3 | 2±7 |
| Medium | 14±10 | 8±11 | 8±12 | 7±9 | 5±8 | 6±8 | 2±4 |
| Large | 20±14 | 10±10 | 11±12 | 9±11 | 5±7 | 4±8 | 3±5 |
| Sex ratio [proportion of males] |  |  |  |  |  |  |  |
| Small | 0.41±0.17 | 0.59±0.24 | 0.52±0.13 | 0.60±0.14 | 0.61±0.29 | 0.69±0.00 | 0.53±0.00 |
| Medium | 0.41±0.25 | 0.55±0.23 | 0.56±0.14 | 0.49±0.23 | 0.54±0.15 | 0.50±0.16 | 0.73±0.05 |
| Large | 0.39±0.20 | 0.56±0.12 | 0.49±0.21 | 0.56±0.12 | 0.54±0.11 | 0.46±0.16 | 0.62±0.15 |
| Hatching success |  |  |  |  |  |  |  |
| Small | 0.64±0.19 | 0.52±0.32 | 0.68±0.17 | 0.60±0.23 | 0.70±0.14 | 0.72±0.00 | 0.77±0.00 |
| Medium | 0.62±0.17 | 0.48±0.32 | 0.49±0.18 | 0.51±0.22 | 0.50±0.31 | 0.54±0.22 | 0.38±0.16 |
| Large | 0.67±0.17 | 0.48±0.21 | 0.56±0.21 | 0.63±0.18 | 0.45±0.25 | 0.50±0.22 | 0.60±0.13 |
| Eclosion time [days] |  |  |  |  |  |  |  |
| Small | 12±1 | 13±2 | 12±1 | 14±3 | 13±1 | 14±0 | 19±0 |
| Medium | 12±1 | 14±2 | 13±2 | 13±2 | 14±2 | 13±1 | 15±1 |
| Large | 12±1 | 14±2 | 14±2 | 14±1 | 15±3 | 15±2 | 14±1 |
| Emergence of first wasp [days] |  |  |  |  |  |  |  |
| Small | 24±1 | 24±2 | 24±1 | 25±1 | 25±2 | 23±1 | 28±1 |
| Medium | 23±2 | 24±2 | 24±1 | 24±2 | 26±2 | 26±2 | 28±3 |
| Large | 24±2 | 25±1 | 25±2 | 26±2 | 25±2 | 25±2 | 24±2 |
| Emergence of last wasp [days] |  |  |  |  |  |  |  |
| Small | 25±2 | 26±2 | 26±2 | 26±2 | 26±2 | 25±1 | 30±1 |
| Medium | 24±2 | 25±2 | 25±1 | 26±2 | 27±2 | 28±1 | 29±2 |
| Large | 25±2 | 26±2 | 27±2 | 27±2 | 26±2 | 26±2 | 25±2 |
| Tibia length of females [mm] |  |  |  |  |  |  |  |
| Small | 0.897±0.056 | 0.861±0.069 | 0.876±0.070 | 0.832±0.093 | 0.893±0.068 | 0.881±0.050 | 0.796±0.038 |
| Medium | 0.885±0.060 | 0.864±0.067 | 0.860±0.061 | 0.868±0.048 | 0.867±0.077 | 0.837±0.051 | 0.767±0.072 |
| Large | 0.882±0.054 | 0.822±0.071 | 0.850±0.062 | 0.850±0.073 | 0.832±0.059 | 0.895±0.068 | 0.876±0.058 |
| Tibia length of males [mm] |  |  |  |  |  |  |  |
| Small | 0.896±0.050 | 0.869±0.066 | 0.889±0.054 | 0.840±0.077 | 0.907±0.046 | 0.912±0.035 | 0.836±0.038 |
| Medium | 0.881±0.051 | 0.863±0.065 | 0.870±0.064 | 0.882±0.042 | 0.868±0.074 | 0.859±0.048 | 0.784±0.055 |
| Large | 0.879±0.058 | 0.832±0.071 | 0.852±0.066 | 0.853±0.073 | 0.864±0.058 | 0.867±0.072 | 0.862±0.071 |
